# An investigation of the bio-medical waste produced in India during the COVID-19 pandemic and Maharashtra state (pre-COVID-19 and post-COVID-19) analysis: a GIS-based approach

**DOI:** 10.1007/s43999-023-00023-9

**Published:** 2023-04-28

**Authors:** Wasim Ayub Bagwan

**Affiliations:** School of Rural Development, Tata Institute of Social Sciences, Tuljapur, 413601 Maharashtra India

**Keywords:** Bio Medical Waste (BMW), COVID-19, COVID-19 related waste, Geographical Information System (GIS), Health care waste

## Abstract

**Background:**

The COVID-19 pandemic exerted substantial pressure on global healthcare systems and facilities, putting the lives of countless individuals at risk. In addition, the treatment of patients during the pandemic resulted in an unprecedented increase in the volume of medical waste generated, including biomedical waste (BMW) or healthcare waste (HCW), which poses a risk of infectious disease transmission. As the second most populous country in the world, India faced a severe challenge in managing its healthcare waste infrastructure during this time (2020–2021). Proper disposal of BMW was of utmost importance to prevent the spread of infectious agents and to safeguard public health.

**Methods:**

The environmental monitoring and management framework of the country is well planned and governed by the Central Pollution Control Board (CPCB), which carefully handles the BMW across the states and union territory of the country. Through the execution of Android based application named ‘COVID19BMW’, India has laid the foundation of identification, classification, data collection, and management regarding the BMW. Further, the temporal scale of BMW generation tracking was further improved from a monthly to a daily basis by using the COVID19BMW tool. This data was used to map the change taken place across the India. Additionally, by using Geographical Information System the BMW is mapped using Choropleth method.

**Results:**

The current study conducted a national-level analysis of BMW generated during the COVID-19 pandemic in India. The results revealed that, in the year 2020, the seven states and the National Capital Territory (NCT) of Delhi generated the highest amounts of BMW, with Gujarat, Maharashtra, Kerala, Karnataka, Tamil Nadu, Uttar Pradesh, and West Bengal being the top BMW generating states. Additionally, the change detection equation was used to map the changes. The investigation analysed the daily changes in BMW generation between 2020 and 2021 at the national level. The results indicated a significant decreasing trend of -50.35% in BMW generation per day. In the case of Maharashtra state, the change detection analysis for the pre-COVID-19 and post-COVID-19 pandemic periods showed an increased trend of approximately 32%. However, in 2021, a decreasing trend was observed, with a -2.23% reduction in BMW generation compared to 2020 on the daily basis of BMW generation. These findings suggest that the COVID-19 pandemic has influenced BMW generation of waste, and the results can provide insights for improving waste management policies and practices.

**Discussion:**

In this study, a Geographical Information System (GIS) was employed to create a mapped representation of the BMW data at national scale. Further, the study investigated changes in BMW generation in Maharashtra state during the COVID-19 pandemic. Analysis of changes in BMW generation revealed a pattern of BMW generation during the pandemic. The use of GIS technology to track these changes proved to be a valuable tool in providing a synoptic view of the overall BMW condition across India and identifying areas where infectious waste poses a significant threat. The visualisation of data using the GIS technique provided an easy means of identifying hotspots of BMW generation, which is more effective compared to a tabular format.

## Introduction

Coronavirus disease (COVID-19) is an infectious disease caused by the SARS-CoV-2 virus. COVID-19 is a contagious illness caused by a novel coronavirus. COVID-19 is the third large zoonotic coronavirus illness outbreak in less than two decades [1]. Due to COVID-19, the pressure on the healthcare system has been increased. Since the year 2020, the outbreak of COVID-19 has created an immense increment in the generation of Bio Medical Waste (BMW). Although BMW represents a relatively small portion of the total waste generated in a community, medical waste management is considered an important issue worldwide [2]. Different countries in the world have different terminologies for medical waste, like health care waste (HCW). According to the World Bank, HCW management is a process to ensure proper hospital hygiene and the safety of health care workers and communities. It includes planning and procurement, construction, staff training and behaviour, proper use of tools, machines, and pharmaceuticals, proper disposal methods inside and outside the hospital, and evaluation [3]. The BMW disposal system in developing countries is quite improper because of lack of availability of treatment and related facilities. Due to the pressure of over-crowded hospitals, workers make mistakes and get infected by them. Adopting the proper management of medical waste inside health facilities, by incineration or sterilising and shredding, can greatly reduce the transmission of infection and the transmission of pathogens [4]. Biomedical waste is not treated in the same way as municipal garbage is. Under the Ministry of Environment, Forest, and Climate Change (MoEFCC), the central pollution control board (CPCB) is the apex body for monitoring the country's BMW management operations. Each state has its own state pollution control board, which monitors and regulates BMW operations in the state and reports findings to the CPCB [5]. Bio-medical waste means any waste which is generated during the diagnosis, treatment or immunization of human beings or animals or research activities pertaining thereto or in the production or testing of biologicals, and including categories mentioned in Schedule I of the bio-medical waste management and handling rules 1998 [6]. And, Health care facility means a place where diagnosis, treatment or immunisation of human beings or animals is provided, irrespective of the type and size of the health treatment system, and research activity pertaining thereto [7]. In the share of BMW, nearly 85% is general, non-hazardous waste comparable to domestic waste. The remaining 15% is contagious, hazardous, and prone to infection with chemical or radioactive properties. The BMW contains pathological, infectious, sharp, chemical, cytotoxic, pharmaceutical, radioactive waste, etc. [8]. BMW is generated in both the government and private sectors in India. The private sector plays an important role in providing services in both rural and urban areas and can play a key role in responding to disease outbreaks and pandemics [9]. It is always in top five health care waste producers. During the COVID-19 pandemic, the highest cases have been recorded in the Maharashtra state, and it is the highest BMW produced in India. Any waste create multiple environmental and administration issue. Here, in case of COVID-19 BMW, the scenario identification across the nation during first and second wave is made crucial to depict the threat of this infectious waste. Thus, the objectives of this study is to determine how much BMW was generated in India during COVID-19 pandemic and map the changes occurred between 2020 and 2021, additionally to make visualization of the whole statistics by using Geographical Information Science (GIS). Moreover, for Maharashtra state the pre-COVID-19 (2018, 2019) BMW is available and was used to differentiate previous condition and during COVID-19 pandemic condition (2020, 2021) with the help of rate of change observed and GIS based map forms.

### At a Glance: India and Maharashtra State

**Fig. 1:**
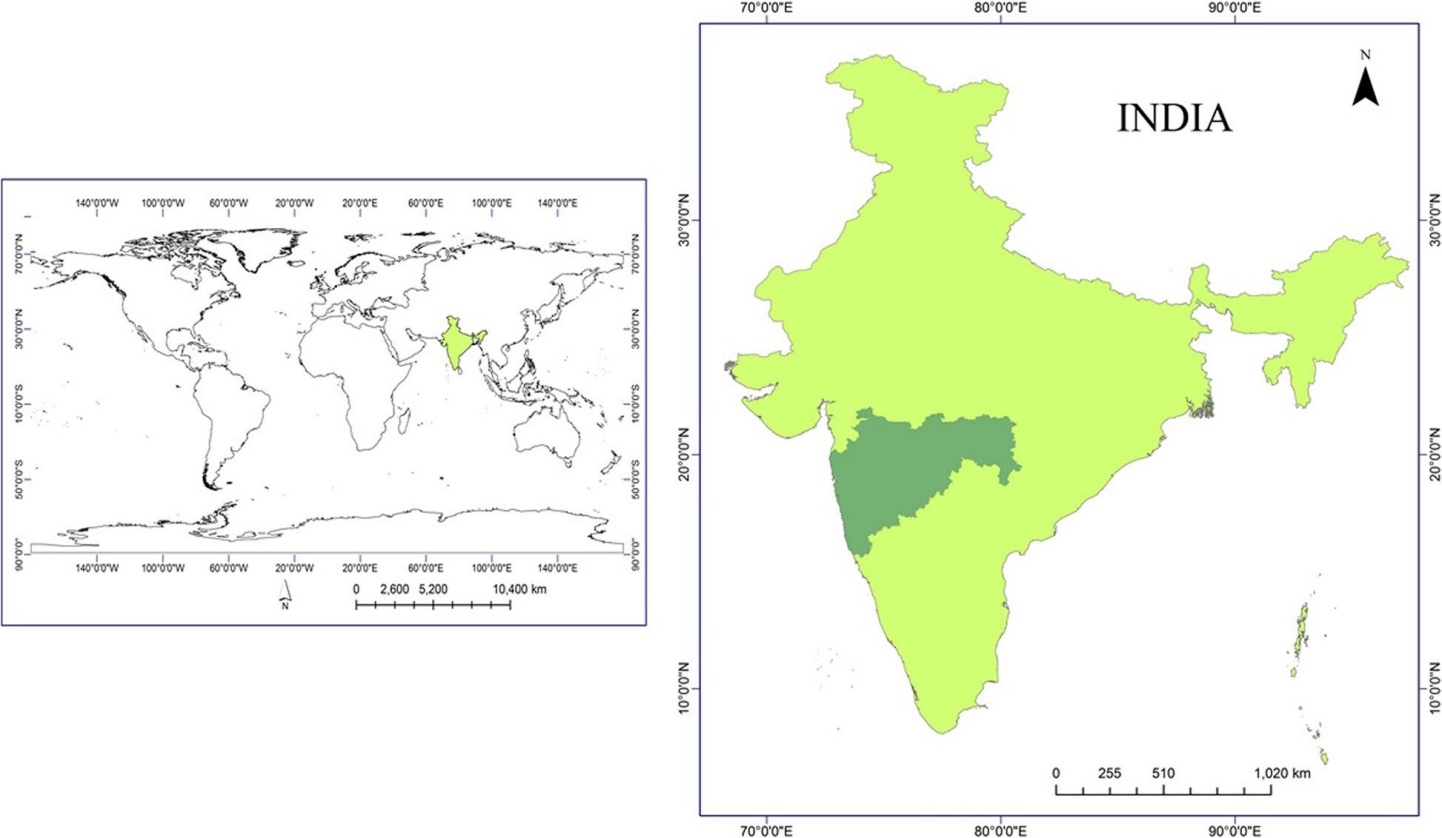
Location of Maharashtra State of India

The location of India and Maharashtra State is displayed in Fig. 1, India is the second most populous country in the world, covering an area of 3.3 million km^2^. The climate of India can broadly be categorised as a tropical monsoon one. But, in spite of much of the northern part of India lying beyond the tropical zone, the entire country has a tropical climate marked by relatively high temperatures and dry winters. There are four seasons: winter (December-February), summer (March-June), the south-west monsoon season (June–September), and the post monsoon season (October–November). From a geographical view, the mainland comprises of four regions, namely the great mountain zone, the plains of the Ganga and the Indus, the desert region, and the southern peninsula [10]. India was the first country in South Asia to establish a legal framework for the management of health care waste. The development of India’s legal framework began in 1995 [11]. Maharashtra State is located in the western and central parts of India and has a 720 km long coastline along the Arabian Sea and is also fortified naturally by the *Sahyadri* and *Satpuda* mountain ranges. For administrative convenience, the state has been divided into 36 districts and six revenue divisions. With a population of 11.24 crore, as per Population Census 2011 and with geographical area of about 3.08 lakh km^2^ the State ranks second by population and third in terms of geographically spatial coverage. Maharashtra is one of the highly urbanised states in India [12].

## Material and methodology

### Data

The BMW data during the COVID-19 period is available on the Central Pollution Control Board website (Source: https://cpcb.nic.in/covid-waste-management/). The data contains the waste generated in each state and union territory of India. Reports from May 2020 to December 2021 are available. The Maharashtra Pollution Control Board (MPCB) published a report on BMW management at district level for the years 2019 and 2020, which can be downloaded from its official website (Link: https://www.mpcb.gov.in/waste-management/biomedical-waste). The Unique Identification Authority of India (UIDAI) projected population data was downloaded from https://uidai.gov.in/images/state-wise-aadhaar-saturation.pdf [Accessed on Feb 25 2022].

### Literature for this study

The scientific literature was gathered from Scopus and Web of Science indexed journals in order to evaluate the state of healthcare waste (also known BMW) during COVID-19 pandemic. Additionally, for recent updates in the advisory, reports on BMW rules, management, treatment, and disposal of COVID-19 waste, the official websites regarding health, pollution control boards, and public domain portals were visited to download the data, tables, or posters.

### Biomedical waste in the current scenario

**Fig. 2:**
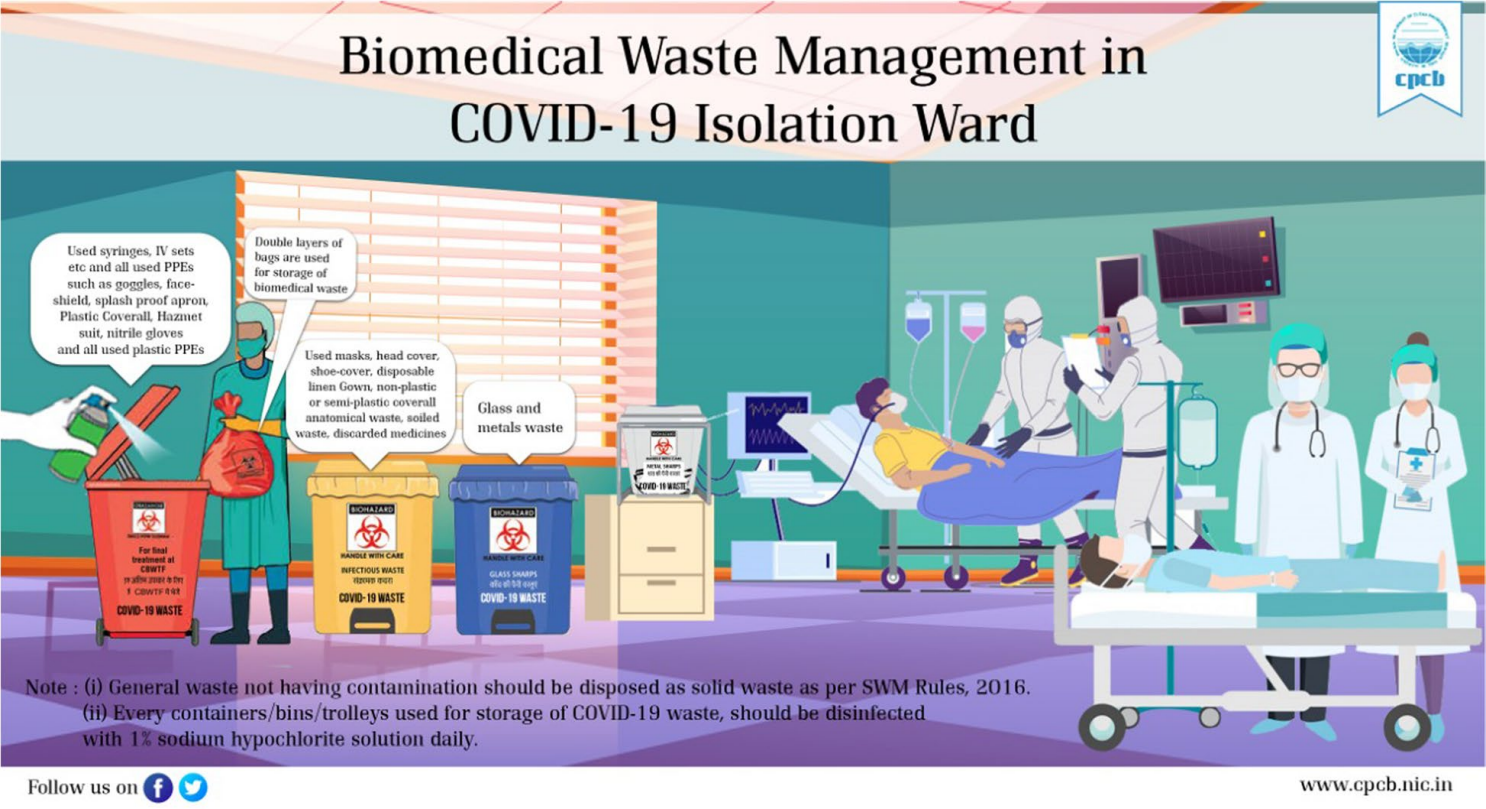
Onsite waste segregation of COVID-19 (Source: https://cpcb.nic.in/uploads/Projects/Bio-Medical-Waste/Poster1_covid.jpg)

**Fig. 3:**
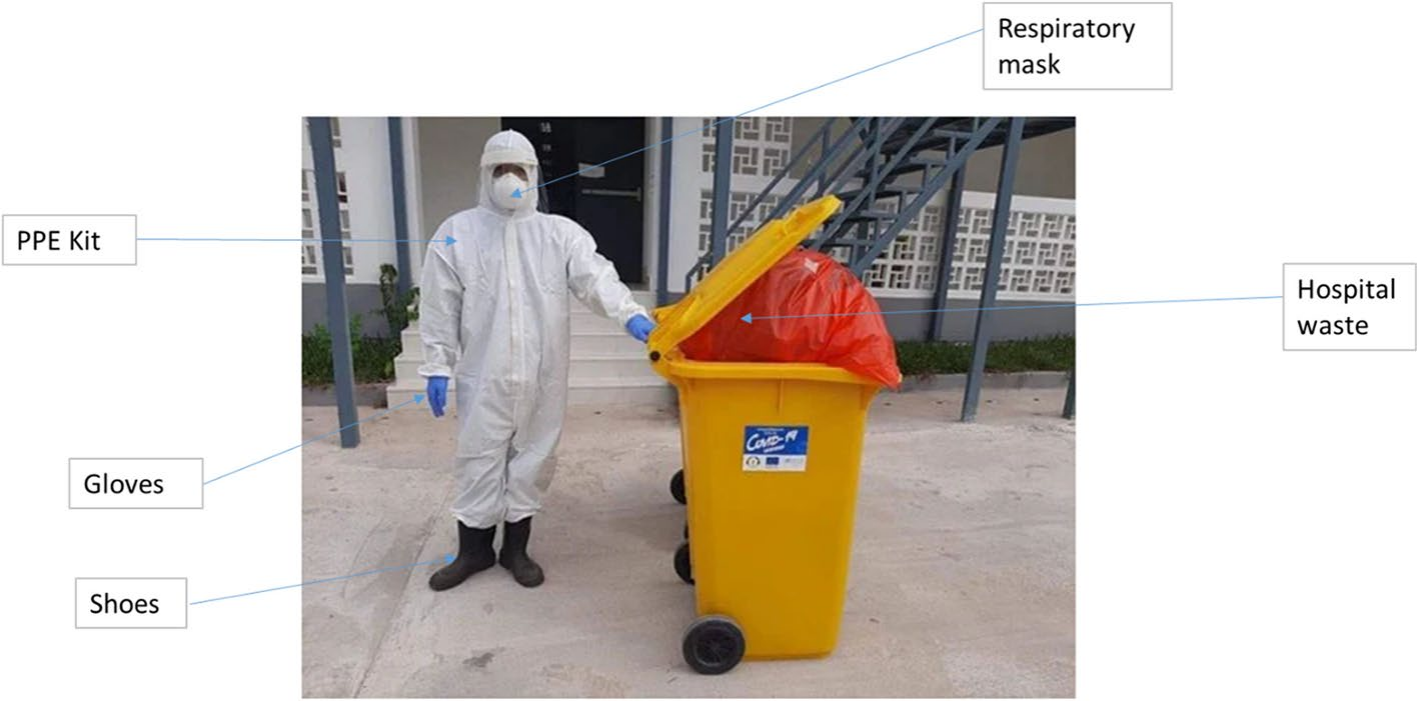
Hazardous waste generated from the response to COVID-19 [18]

The general flow of BMW life cycle in India is the cradle-to-grave approach is utilized to manage BMW, encompassing the characterization, quantification, segregation, storage, transportation, and treatment of the waste [13]. Lack of proper management of waste places a heavy load on ecosystems and endangers public health. All waste management techniques come with trade-offs for the environment, such as air pollution from incineration, methane emissions from landfills, or the energy costs of transporting recyclables over vast distances (often internationally) to central facilities [14]. In the COVID-19 global pandemic, modification to existing waste facilities to control the unusual medical waste and its associated viral spread effect requires proper information on the amount of biomedical waste generated, hot spots for waste generation and available treatment facilities [15]. Globally, as a result of COVID-19, there has been an increase in the production of BMW; this has led to serious concerns about its management. Processing centres were overloaded by such waste, through the departments of such centres, food waste was also included as part of infectious COVID-19 waste [16]. Because the bulk of personal protective equipment (PPE) goods are designed for single use, the increased use and demand for medical grade and non-medical (or civil use) PPE has worsened the already significant worldwide waste management dilemma. BMW, including PPE, was commonly cremated prior to the epidemic. The PPE used by an expected 5 billion extra individuals adds to the waste volume currently created and disposed of by incineration. Without a stronger end-of-life plan, plastic garbage is expected to quadruple in volume over the next 20 years, with the quantity entering the world's seas quadrupling [17]. Further, the CPCB has timely provided the guidelines for the segregation of COVID-19 related waste. Figure 2 shows the post representing medical waste segregation at the isolation ward level. Also, hazardous waste generated during COVID-19 treatment at a health care facility is portrayed in Fig. 3 [18]. WHO has provided the COVID-19 related healthcare waste generated and its potential threat of infection in Table 1 [19].

**Table 1:** Main types of COVID-19 related healthcare waste [19]

Item	Type of waste	Requires safe handling and treatment
Mask	Infectious	Yes
Gloves	Infectious	Yes
Gown	Infectious	Yes
SARS-CoV-2 Rapid Antigen Test (RAT)	Non hazardous	Most components are recyclable; a very small volume of reagent may require safe handling and disposal if dealing with large numbers of tests
PCR testing cartridge	Chemical	Yes (contains guanidinium thiocyanate)
Vaccine vial	Non hazardous	No
Vaccine needle	Sharps	Yes (packaging material is recyclable)
Plastic packing and containers	Non hazardous	No

### India’s laws and legislation

The rules framed by the Ministry of Environment and Forests (MoEF), Govt. of India, known as the "Bio-Medical Waste (Management and Handling) Rules, 1998, notified in July 1998, provide uniform guidelines and a code of practise for handling health care waste. In March 2016, the Ministry of Environment, Forest and Climate Change (MoEFCC) Bio Medical Waste Management Rules, 2016 were revised. Biomedical waste categories and their segregation, collection, treatment, processing, and disposal options were documented in BMW Rules 2016 as attached in the supplementary material.

### Tracking of biomedical waste through an android application

**Fig. 4:**
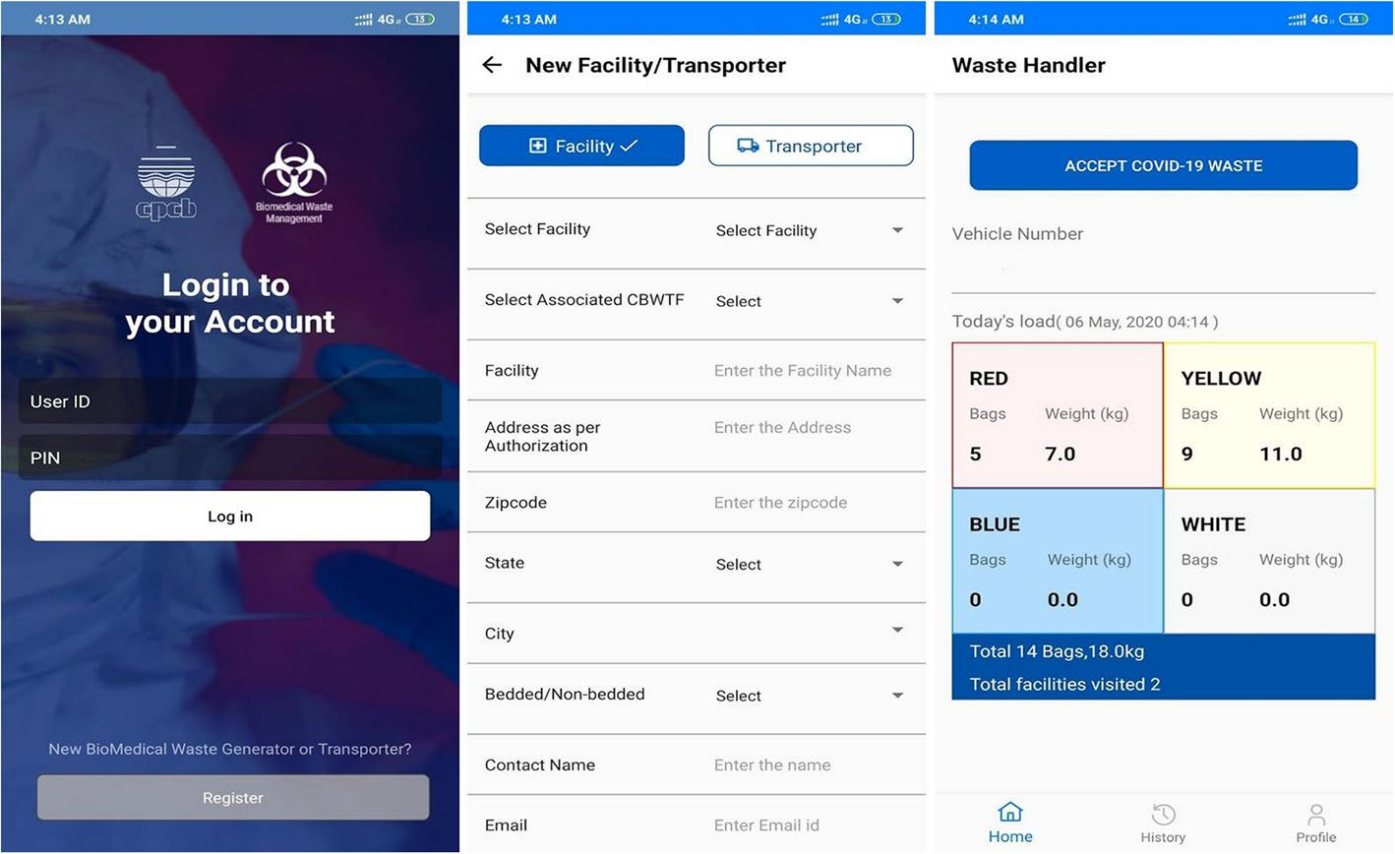
COVID19BMW Android Application by CPCB

"COVID19BWM" is a software application for tracking the generation, collection, and disposal of COVID-19 bio-medical waste at various health care facilities and hospitals (HCF), quarantine centres, isolation wards, testing labs, covid-19 sample collection centres, and urban local bodies involved in waste collection from home quarantine centres and homecare units. This application will allow information to be shared across many stakeholders [20]. A web-based application is also available for the authorities to monitor BMW. Figure 4 shows an Android-based application to collect the BMW generated during COVID-19 pandemic—COVID19BMW.

### Monitoring and treatment of BMW during COVID-19

In addition to the health problems, COVID-19 has a significant long-term impact on our environment, which will in turn have a wide impact on all life forms. Although our main concerns right now are health-related, widespread misunderstanding of environmental issues will lead to many more deaths in the future [21]. A healthcare waste management strategy refers to a facility's programme for managing generated waste for disposal. It always addresses: (1) compliance with guidelines; (2) responsibilities of staff members; (3) definitions/classification of healthcare waste; (4) specific procedures for handling healthcare waste; and (5) training of related workers. Different countries have adopted different strategies in response to the management of the large and contagious amount of waste produced during the COVID-19 pandemic [22]. The Biomedical Waste Management Rules, 2016 and the guidelines issued by the CPCB require common BMW treatment facilities (CBWTFs) to operate in accordance with the standards notified under the Biomedical Waste Management Rules, 2016. State Pollution Control Boards or Pollution Control Committees are the authorities in charge of enforcing the rules and ensuring compliance. BMW generated at state level in India in the year 2020 (tonne/month) is shown in Table 2, and BMW generated in the year 2021 (tonnes/day) is shown in Table 3. In the current situation of pandemics, the public sector is working hard to strengthen infrastructure and limit such events in order to reduce environmental pollution and ensure the timely disposal of BMW, particularly COVID-19-related trash [21].

**Table 2:** Bio Medical waste generated at state level in India in tonnes per month  in Year 2020

Indian States and Union Territories	Total Population (Projected 2020)	June	July	August	September	October	November	December	Mean BMW in Tonnes/month	Mean BMW in Tonnes/Day
Andaman & Nicobar^*^	4,17,036	0.42	INP	INP	0.42	0.434	0.42	0.43	0.42	0.01
Andhra Pradesh	5,39,03,393	165.48	182.81	118.82	112.35	116.095	317.91	328.51	191.71	6.39
Arunachal Pradesh^*^	15,70,458	3.36	3.36	3.8	3.36	3.472	3.36	3.47	3.45	0.12
Assam	3,56,07,039	28.38	20.68	12.57	62.61	51.739	50.07	23.41	35.64	1.19
Bihar	12,47,99,926	6.84	20.76	41.54	45.36	44.64	28.08	23.31	30.08	1.00
Chandigarh	11,58,473	29.85	5.65	55.34	43.02	73.191	70.83	73.19	50.15	1.67
Chhattisgarh	2,94,36,231	11.19	INP	13.39	9.3	9.61	9.3	9.61	10.40	0.35
Daman and Diu^**^, Dadra and Nagar Haveli^**^	6,15,724	0	INP	0	0.48	2.387	1.08	1.15	0.85	0.03
NCT of Delhi	1,87,10,922	333.42	389.58	296.14	382.5	365.893	385.47	321.32	353.47	11.78
Goa^*^	15,86,250	0.81	0.81	INP	15	7.75	5.43	5.39	5.87	0.20
Gujarat	6,38,72,399	350.79	306.14	360.04	622.89	545.879	423.51	479.57	441.26	14.71
Haryana	2,82,04,692	75.33	184.18	210.69	278.31	238.452	239.4	209.93	205.18	6.84
Himachal Pradesh	74,51,955	3.81	12.5	4.94	25.2	28.117	30.03	48.24	21.83	0.73
Jammu and Kashmir	1,38,95,343	10.71	9.77	51.77	57.39	59.303	44.82	35.12	38.41	1.28
Jharkhand	3,85,93,948	INP	INP	2.59	4.8	4.96	4.8	11.63	5.76	0.19
Karnataka	6,75,62,686	84	540.28	588.03	168	218.023	210.99	218.02	289.62	9.65
Kerala	3,56,99,443	141.3	293.32	588.05	494.1	641.979	600.39	542.47	471.66	15.72
Lakshadweep	73,183	0.3	INP	INP	0.3	0.31	0.3	0.31	0.30	0.01
Madhya Pradesh	8,53,58,965	224.58	56.4	106.59	339	308.419	208.65	249.49	213.30	7.11
Maharashtra	12,31,44,223	524.82	1180	1359	524.82	542.314	609	629.3	767.04	25.57
Manipur	30,91,545	5.13	0.2	2.09	5.13	5.301	5.13	9.27	4.61	0.15
Meghalaya	33,66,710	5.1	1.74	6.34	9.9	12.028	7.65	8.56	7.33	0.24
Mizoram^*^	12,39,244	4.2	INP	INP	4.2	3.224	3.12	3.22	3.59	0.12
Nagaland^*^	22,49,695	3.6	3.4	3.1	2.85	3.317	1.86	2.29	2.92	0.10
Odisha	4,63,56,334	31.86	106.63	109.19	134.01	183.458	222.66	125.58	130.48	4.35
Puducherry	14,13,542	18.63	35.82	41.54	63	58.652	28.74	17.11	37.64	1.25
Punjab	3,01,41,373	48	35.59	21.19	234.42	149.606	96.51	86.99	96.04	3.20
Rajasthan	8,10,32,689	177	7.15	50.43	145.08	171.554	141.93	105.93	114.15	3.81
Sikkim	6,90,251	6	0.2	0.3	6	4.216	3.69	2.45	3.27	0.11
Tamil Nadu	7,78,41,267	312.3	401.29	481.1	543.78	524.179	300.75	251.22	402.09	13.40
Telangana	3,85,10,982	12.3	10.5	24.04	188.82	144.801	103.89	68.82	79.02	2.63
Tripura	41,69,794	0.45	INP	INP	0.45	0.465	0.45	0.47	0.46	0.02
Uttarakhand	1,12,50,858	0.45	0.82	41.85	21.72	108.996	56.76	76.26	43.84	1.46
Uttar Pradesh	23,78,82,725	210	307.54	408.86	507.15	478.082	316.71	276.46	357.83	11.93
West Bengal	9,96,09,303	195	136.37	235.12	434.76	486.793	330.84	279.06	299.71	9.99
Total	137,05,08,600	3025.41	4253.49	5238.45	5490.48	5597.639	4864.53	4527.56		

INP Information Not Provided, As per the data availability and understanding purpose, population of Ladakh (Union Territory) is merged in Jammu & Kashmir (UT)), The BMW of two separate UT i.e. Dadra & Nagar Haveli and Daman & Diu is merged

^*^As per earlier information provided by States/UTs

^**^Using CBWTF, Surat, Gujarat for disposal of biomedical waste

**Table 3:** Bio medical waste generated at state level in India in year 2021

Indian States and Union Territories	Jan to May	June	July	August	September	October	November	December	Mean BMW (TPD)	Change detection between 2020–2021
	Monthly BMW produced in Tonnes Per Day (TPD)									
Andaman and Nicobar^*^	0.014	0.003	0.08	0.01	0.02	0.02	0.01	0.01	0.02	47.42
Andhra Pradesh	9.99	11.96	5.31	3.84	4.04	2.27	1.15	0.74	4.91	-23.13
Arunachal Pradesh^*^	0.112	0.003	0	0	0	0	0	0	0.01	-87.52
Assam	0.52	0.81	0.45	0.5	0.23	0.17	0.16	0.17	0.38	-68.33
Bihar	1.06	1.19	0.32	0.12	0.13	0.12	0.1	2.48	0.69	-31.17
Chandigarh	1.91	1.78	1.44	0.42	0.26	0.27	0.26	0.21	0.82	-51.02
Chhattisgarh	2.76	1.02	0.38	0.2	0.11	0.11	0.09	0.09	0.60	71.63
Daman & Diu, Dadra & Nagar Haveli^**^	0.065	0.039	0	0	0	0	0	0	0.01	-54.09
NCT of Delhi	18.79	7.68	3.55	2.37	1.56	0.89	0.9	1.03	4.60	-60.99
Goa^*^	0.45	0.88	0.5	0.37	0.27	0.2	0.18	0.18	0.38	93.73
Gujarat	21.98	5.48	1.51	0.77	0.74	0.77	0.52	0.53	4.04	-72.55
Haryana	13.11	5.04	1.94	1.38	1.31	0.54	0.47	0.58	3.05	-55.46
Himachal Pradesh	2.27	2.14	0.41	0.41	0.37	0.29	0.23	0.22	0.79	8.89
Jammu and Kashmir	2.49	1.82	0.83	1.49	0.58	0.28	0.41	0.49	1.05	-18.09
Jharkhand	0.56	0.83	0.1	0.02	0	0	0	0.01	0.19	-0.97
Karnataka	16.91	14.5	7.11	4.35	3.18	1.63	1.27	1.12	6.26	-35.17
Kerala	23.71	26.95	20.42	17.9	17.73	11.16	7.24	5.8	16.36	4.08
Lakshadweep	0.01	0.01	0	0	0	0	0	0	0.00	-75.33
Madhya Pradesh	7.32	4.18	0.47	0.46	0.44	0.32	0.49	0.4	1.76	-75.25
Maharashtra	19.02	14.25	8.31	6.64	5.3	3.79	2.67	2.93	7.86	-69.24
Manipur	0.13	0.31	0.07	0.14	0.03	0.03	0.02	0.01	0.09	-39.77
Meghalaya	0.25	0.6	0.58	0.5	0.51	0.2	0.16	0.4	0.40	63.69
Mizoram^*^	0.033	0.12	0.14	0.16	0.14	0.13	0.14	0.14	0.13	4.69
Nagaland^*^	0.074	0.074	0	0	0	0	0	0	0.02	-80.97
Odisha	6.65	9.14	3.98	2.7	2.82	1.72	1.4	0.53	3.62	-16.83
Puducherry	1.81	1.67	0.74	0.44	0.45	0.24	0.22	0.17	0.72	-42.82
Punjab	4	3.87	1.09	0.77	0.62	0.46	0.72	0.34	1.48	-53.65
Rajasthan	4.98	3.61	0.45	0.27	0.19	0.18	0.15	0.11	1.24	-67.35
Sikkim	0.015	0.21	0.3	0.33	0.15	0.13	0.11	0.09	0.17	53.32
Tamil Nadu	13.57	26.04	6.41	4.6	4.14	3.49	3.17	2.62	8.01	-40.27
Telangana	4.96	5.24	1.65	1.51	1.17	0.72	0.54	0.42	2.03	-23.08
Tripura	0.02	0.062	0.04	0.04	0.03	0.01	0.11	0.01	0.04	164.22
Uttarakhand	1.98	1.2	0.43	0.21	0.11	0.04	0.04	0.05	0.51	-65.27
Uttar Pradesh	15.91	4.38	1.81	1.37	1.33	1.21	0.59	0.34	3.37	-71.77
West Bengal	5.72	7.06	1.96	1.27	1.15	0.91	1.18	0.81	2.51	-74.90
	203.153	164.151	72.78	55.56	49.11	32.3	24.7	23.03	2.23	

*INP* Information Not Provided, As per the data availability and understanding purpose, population of Ladakh (Union Territory) is merged in Jammu & Kashmir (UT)), The BMW of two separate UT i.e. Dadra & Nagar Haveli and Daman & Diu is merged

^*^As per earlier information provided by States/UTs

^**^Using CBWTF, Surat, Gujarat for disposal of biomedical waste

### Using Geographical Information System (GIS)

The COVID-19 pandemic, there emerges need of collaborative geospatial infrastructure to understand the situation in a better way at various levels. Increasing need of real-time maps, location-wise information to monitor the data along with data analytical tools has significant role in providing information to its stakeholder [23]. With the help of the Geographical Information System (GIS), we can prepare thematic maps. Thematic maps focus on a particular theme or topic. A Choropleth map is also a kind of thematic map in which maps contain areas that are shaded or patterned in proportion to the statistical variable being displayed on the map. Data is aggregated over predefined areal units. It will be best fitted while using it with standardisation [24]. For the current study, a shape file of Indian states and districts of Maharashtra state has been engaged and provided the attribute data as shown in Tables 3 and 4. The quantile classification approach equalises the quantity of values for data classes at the extremes and the middle. On the map, each class is evenly represented, and calculating the classes is simple. For ordinal data, quantile classification is also highly helpful [25].

**Table 4:** Bio medical waste generated in Maharashtra State from January to December of 2020 and 2021

District name	BMW (kg/day) in 2020	BMW (kg/day) in 2021	District name	BMW (kg/day)	BMW (kg/day) in 2021
Ahmednagar	2151	3120	Nanded	927	956
Akola	728	584	Nandurbar	390	452
Amravati	854	698	Nashik	4350	4350
Aurangabad	2710	2835	Osmanabad	558	415
Beed	569	548	Palghar	1155	1181
Bhandara	374	303	Parbhani	223	223
Buldhana	321	490	Pune	11,068	12,580
Chandrapur	978	1050	Raigad	3366	2763
Dhule	560	545	Ratnagiri	472	622
Gadchiroli	326	450	Sangli	1141	1166
Gondia	299	382	Satara	1823	2256
Hingoli	158	177	Sindhudurg	395	537
Jalgaon	1425	1425	Solapur	2141	2147
Jalna	252	274	Thane	13,463	10,118
Kolhapur	1841	2400	Wardha	483	482
Latur	1147	1250	Washim	270	200
Mumbai	16,450	16,658	Yavatmal	677	481
Mumbai suburban	4711	2819			
Nagpur	3355	3377			

### Bio-Medical waste management in Maharashtra State

MPCB has begun issuing authorization for the disposal of BMW to health care establishments (HCEs) in accordance with the Bio-Medical Waste Management Rules, 2016 [7]. The details of medical waste generated in Maharashtra State from 2020 to 2021 are shown in Table 4 [26, 27]. To compute the change before the COVID-19 pandemic and after the COVID-19 pandemic in the situation of waste generated, the following formula is used to quantify the change in %.

1 $$\text{Rate of change }\left(\mathrm{\%}\right)= (\mathrm{b}-\mathrm{a})/\mathrm{a}) \times 100$$

a = BMW generated last year; b = BMW generation in the current year;

### BMW management and its relationship with the sustainable development goals and the circular economy

Any kind of waste poses a threat to components of the environment. BMW is hazardous and contagious and can be dangerous to animals and humans as well as water, land, and air if mishandled. The proper handling, treatment, and disposal of waste generated in health care facilities protects people, animals, and the environment. In September 2015, the United Nations Assembly, with 193 committee members, set 17 Sustainable Development Goals (SDGs). Health care waste management is closely linked with SDG 3: Good Health and Well-being, SDG 6: Clean Water and Sanitation, SDG 8: Decent Work and Economic Growth, and SDG 12: Sustainable Consumption and Production [28]. Also, a study conducted by Singh et al. [29] shows that there is an opportunity for sustainable resource recovery and recycling of plastic materials from medical waste. And, to avoid catastrophic accumulation of infectious waste during and after pandemics, it is crucial for all countries to implement environmentally sustainable management of medical waste. This will not only add value to the circular economy framework, but will also help reduce emissions.

## Results and discussion

### Application-based monitoring with COVID-19BMW

This application represents a ground-breaking development in the field of real-time waste management. It allows for a more comprehensive understanding of waste generation at various stages, including but not limited to waste generators, waste handlers, and centralized biomedical waste treatment facilities (CBWTFs). The collected data is categorized in accordance with the 2016 BMW management guidelines, facilitating segregation at the point of generation and informing treatment decisions based on the type of waste. This approach significantly reduces the time required for waste segregation during treatment. The application collects data at a national level, enabling real-time tracking of waste generation, flow, identification of hotspots, and informing decision-making regarding necessary future actions. Monitoring the flow of waste generated from a multitude of sources is a critical component of identifying hotspots. The COVID19BWM application has played an instrumental role in monitoring waste generation and identifying the need for additional CBWTFs for safe disposal and treatment. The collected data is highly reliable and can be used to create location, district, and state level maps. The data collected using this application is displayed in Tables 2, 3 and 4.

### GIS-based map analysis of India

**Fig. 5:**
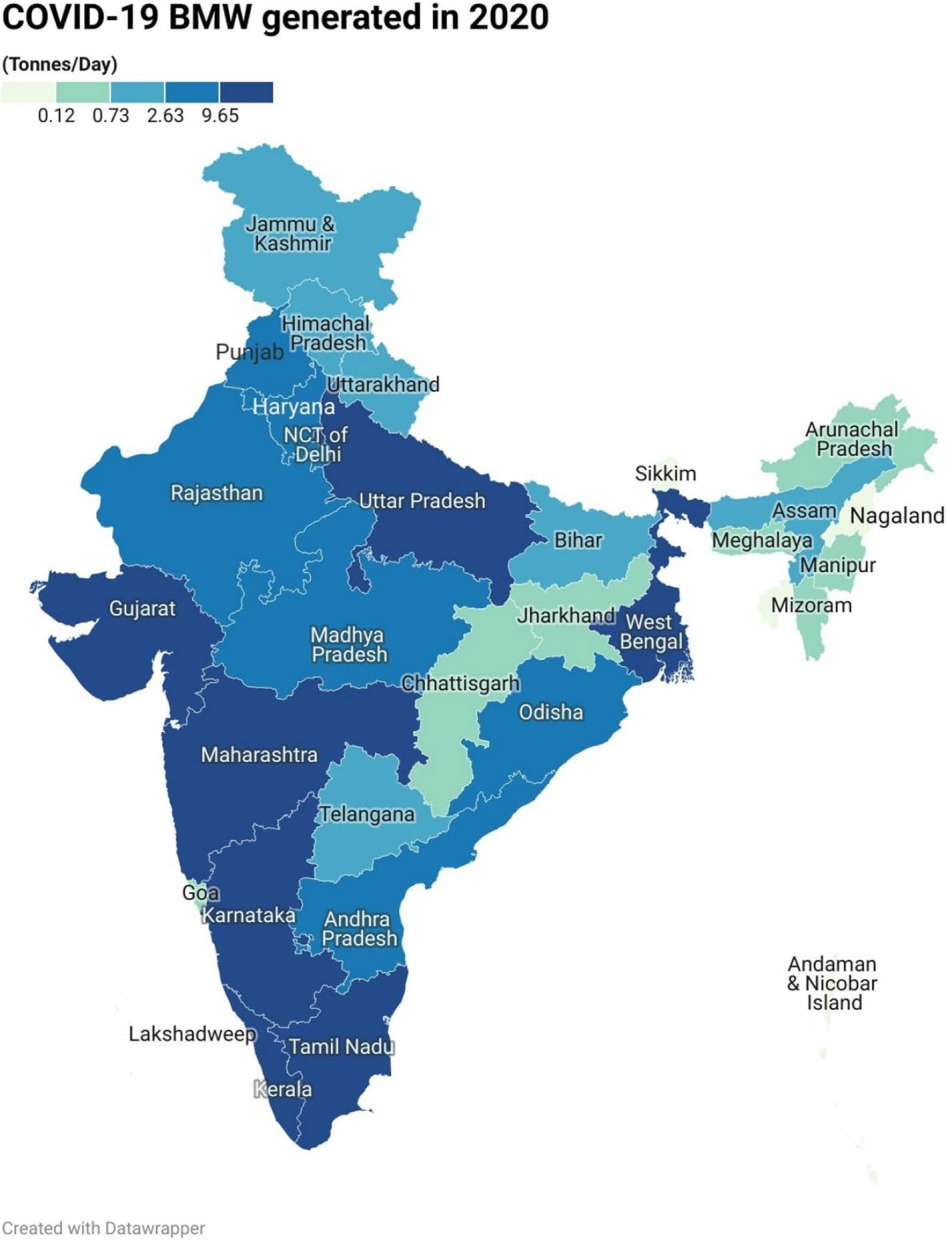
Bio medical waste generated in India in the year 2020 (Tonnes/Day)

**Fig. 6:**
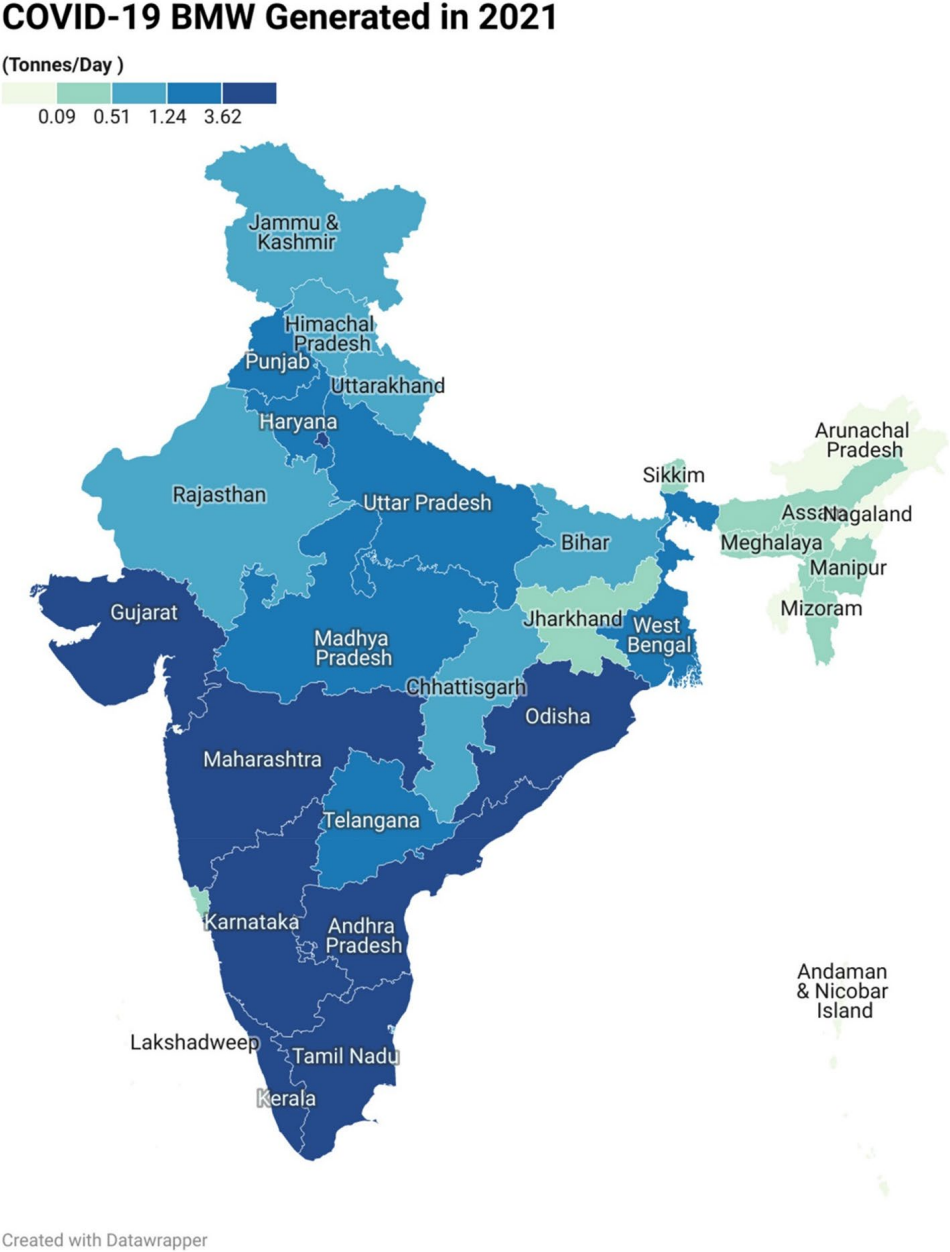
Bio Medical Waste Generated in India in year 2021 (Tonnes/Day)

**Fig. 7:**
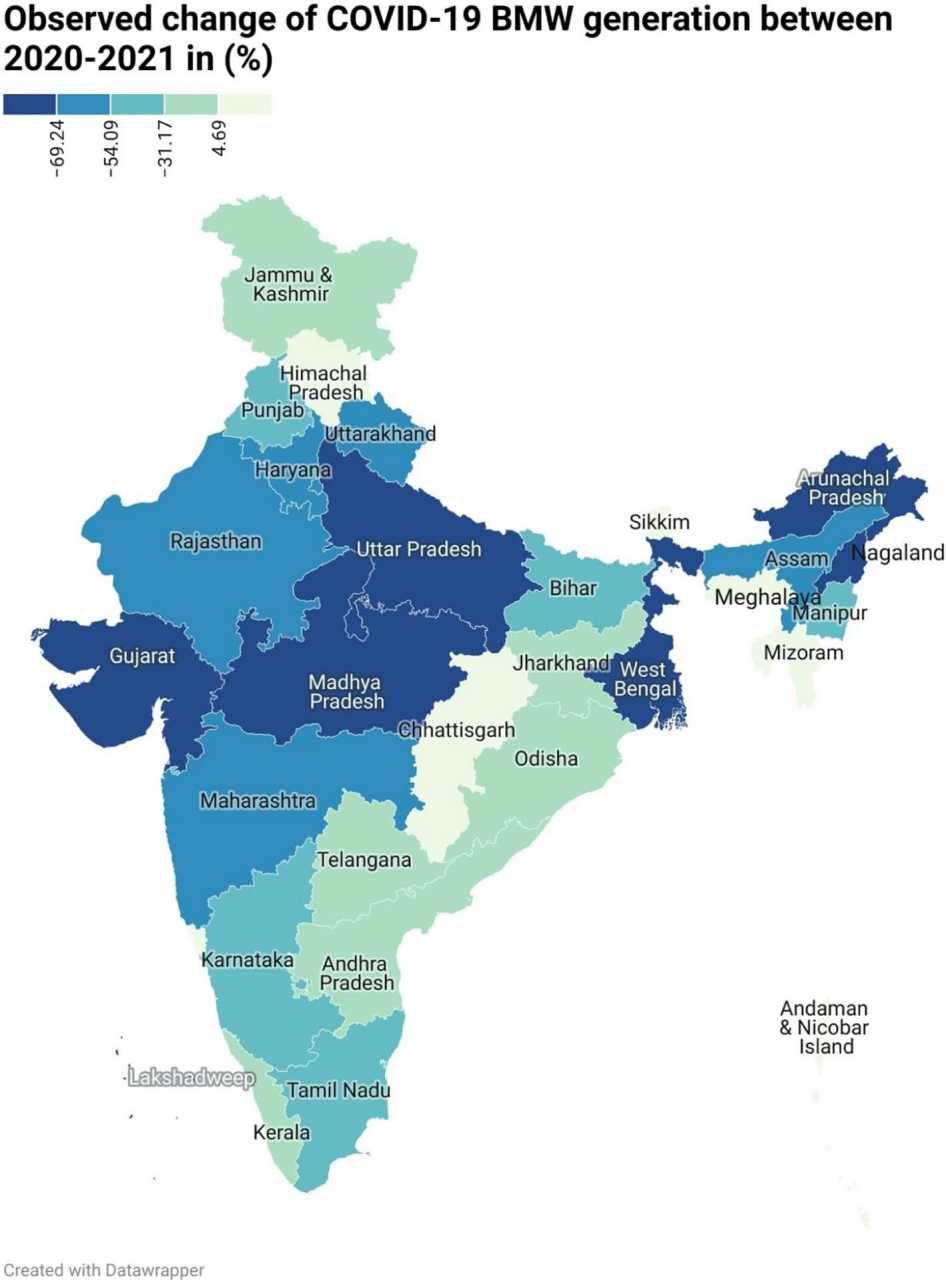
Change detection in BMW generation observed from 2020–2021

To map the BMW generated during the year 2020, numerical data in tonnes/month is collected as a secondary data. And, for sake of understanding and to tune with the BMW generated in the year 2021 the sum is divided by 30 to make uniform arrangement in data at states and Union Territories (UT) level. In India, all states and UTs have been mapped using a GIS platform. The entire BMW data-based thematic map is prepared for India. This powerful visualisation technique overcomes the lacuna of histograms of spatial allocation. According to data released by NDTV on September 18, 2020, the country produces a significant volume of COVID-19-related biological waste (over 100 tonnes per day) [5]. Based on the data shown in Table 2, the average has been calculated and used to prepare the map. Figure 5 highlights the BMW generated in India in the year 2020 that divided into five classes as very low, low, moderate, high and very high; it was prepared using Table 2. Quantile (equal count) based categorization is used to get the very low (0.00–0.12 tonnes/day), moderate (0.73–2.63 tonnes/day) and very high (> 9.65 tonnes/day). For easy understanding we will discuss only consider (very low, moderate and very high) categories can be found in the map i.e. Fig. 5. From the visualization, very high BMW generation has been observed in Gujarat, Kerala, Maharashtra, New Delhi, Karnataka, Tamil Nadu, Uttar Pradesh and West Bengal. In the moderate category, the states such as Bihar, Himachal Pradesh, Uttarakhand, and Telangana were undergone. The North Eastern states, as well as other states and union territories, are among the lower categories of BMW producers. In addition to this, the map for the waste available in the year 2021 shows Data available in Table 3 is used to create the map as shown in Fig. 6. Here, the map created the same method used to prepare Fig. 5 and classified them into five groups. Day-wise data from the year 2021 was available. The average value of each state was calculated and used to map the daily BMW generated in the year 2021. The BMW is categorised as very low (0– 0.09 tonne/day), moderate (0.51– 1.24 tonnes/day) and very high (> 3.62 tonnes/day). From the visual interpretation, it is clear that Kerala, Maharashtra, and Tamil Nadu continue to generate a high volume of BMWs (> 3.62 tonnes/day), additionally Odisha and Andhra Pradesh and Gujarat has shown incremental trend in its BMW generation in 2021. Rajasthan, Chhattisgarh, Bihar and Northern states were contributors of moderate (2.31–6.0 tonnes/day) generators. The high population density states like Uttar Pradesh and Punjab, Haryana, West Bengal has found as a part of high (> 1.24 – 3.62 tonnes/day) category. The North Eastern states has produced medical waste under the category of low (0.09–0.51 tonnes/day) and very low. By comparing the average BMW generated per day in the year 2020 and 2021 it has been found that in first wave of COVID-19, there was 4.49 Tonnes Per Day (TPD) BMW generated which was fallen down to 2.23 TPD in the second wave of COVID-19 in the year 2021. As shown in Table 3, and based on the Eq. 1, the change detection column is prepared to show the difference between 2020 and 2021. Figure 7 shows the observed change in BMW generation from 2020 to 2021, from this analysis it is clear that, there occurred reduction in BMW generated in the year 2021. The average change from 2020 to 2021 was -50.35% in generation of COVID-19 related BMW at national level. According to Fig. 7, we can infer that, NCT of Delhi, Arunachal Pradesh, Maharashtra, Gujarat, Madhya Pradesh, Nagaland, Uttar Pradesh, West Bengal and Lakshadweep (UT), has decreasing trend whereas the states such as Tripura, Goa, Andaman & Nicobar Island (UT), Chhattisgarh, Himachal Pradesh, Meghalaya has doubled the amount of BMW generated as compared to 2020 and shown drastic change. The spatial mapping technique has been found to be robust in visual identification and classifying the shifts in changes that occurred therein.

### COVID-19 Waste generated in maharashtra state

**Fig. 8:**
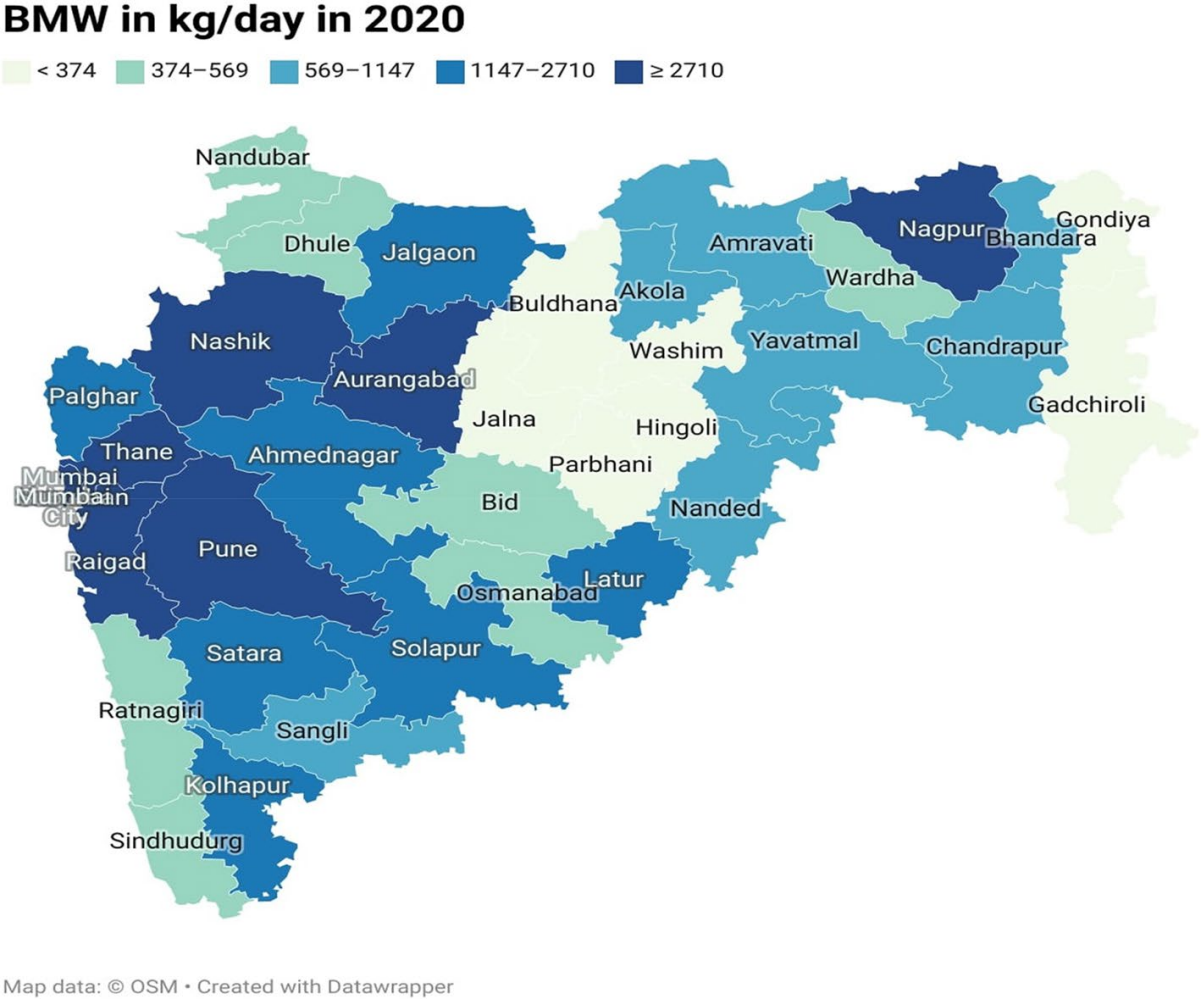
Bio medical waste generated in Maharashtra State in year 2020

**Fig. 9:**
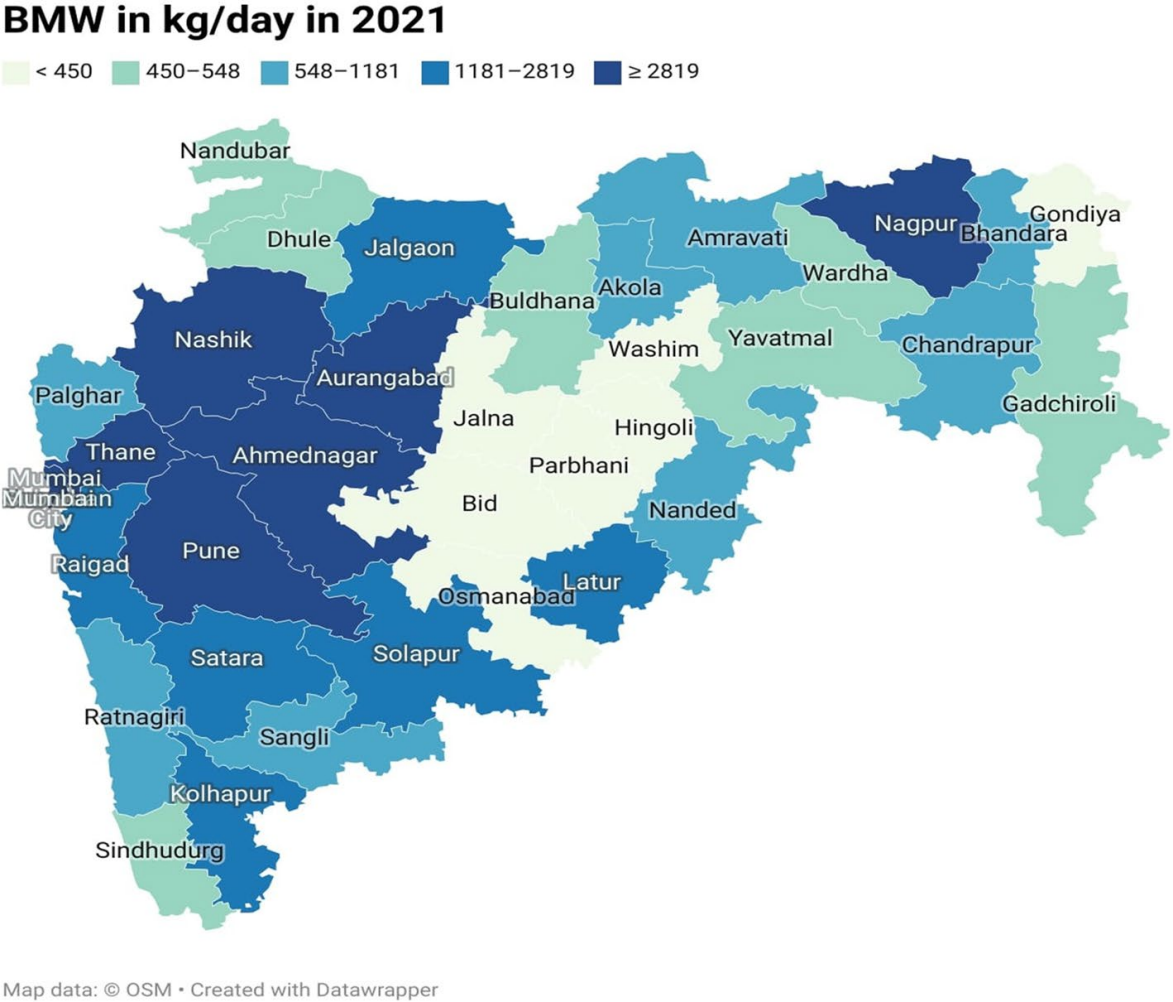
Bio medical waste generated in Maharashtra State in year 2021

Maharashtra state was one of the most COVID-19 infected states and as a second most populous state hence focused for mapping the BMW. Maharashtra was responsible for a significant 17% of India's COVID-19 biomedical waste [30]. District-wise data is available on the MPCB portal and for sake of understanding and based on the quantity, instead of tonnes/day, here we will consider converting into kg/day has been used to map the BMW generation. The numerical digits shown in the Table 4 have been used to map the BMW generated at district level. Figures 8 and 9 represents BMW generated in Maharashtra state in the year 2020 and 2021 respectively. By examining the 2020 BMW generation condition and from map, we can identify that the western part of the state that includes districts viz. Mumbai and its suburban, Pune, Thane, Raigad, Nashik, and Nagpur was a major contributor of very high (> 2710 kg/day) and the moderate waste (569–1174 kg/day) generating districts are the districts surrounding to the high waste generating districts and the very low BMW (< 374 kg/day) are mainly observed in the central and vidarbha region of the Maharashtra state. And, in the year 2021,the COVID-19 BMW waste generation condition in Maharashtra is computed using quantile method shows very high waste (> 2819 kg/day). This shows that very high BMW producers were districts viz. Mumbai with suburban, Pune, Thane, Aurangabad, Nashik, Ahmednagar, Nagpur. In the moderate waste (548–1181 kg/day) generating districts are again found to be neighbouring districts of the high waste generator. In very low waste category (< 450 kg/day) districts are found as in steady condition of 2020 such as Hingoli, Jalna, Parbhani and Washim.

### Change analysis of BMW in Maharashtra State

The value-based change identification showcases the scenario or fluctuations in BMW origination. The detection of changes in BMW data from 2018 to 2021 was considered. Table 5 shows the details of BMWs generated in kilogrammes per day (kg/day). In 2019, there occurred negative change that is -0.26% of waste in HCFs, while there was positive changes were found showing value 5.35% of BMW is generated. As COVID-19 pandemic progressed, there was a 31.95% drastic change in BMW generation. And, based on the BMW produced in 2021, there found -2.23% reduction in BMW observed at state level. The average BMW produced at every day in the state was 2230.94 kg/day in 2021. As compared to 2020, there occurred -2.18% reduction in the average BMW as it was produced in 2021.

**Table 5:** Bio medical waste generated in maharashtra state from 2018–21

Year	BMW Generation	Total no. of Health Care Facilities (HCF)	Change in HCF (%)	Generated BMW in (kg/day)	Occurred Change in (%)
2018	Quantity of Bio-medical Waste Generation (in kg/day)	60,410	-	62,418	-
2019	Quantity of Bio-medical Waste Generation (in kg/day)	63,642	5.35	62,254.62	-0.26
2020	Quantity of Bio-medical Waste Generation (in kg/day)	64,266	0.98	82,146.35	31.95
2021	Quantity of Bio-medical Waste Generation (in kg/day)	64,989	1.13	80,314	-2.23

### BMW’s future prospects

Modern technological intervention by pyrolysis and gasification is emerging as novel and eco-friendly techniques used as thermochemical treatment of wastes as well as energy recovery. For example, plasma gasification and pyrolysis represent a state-of-the-art solution for sustainable management of BMW [31]. Through the segregation of waste, the degradable and recyclable waste can be calculated, which can be further utilised for energy generation purposes. The collected data during the COVID-19 pandemic has the ability to indicate the relationship between the population, area and BMW generation. Such new data gathering can be implemented in developing countries for the assessment of health care waste. The Central Mechanical Engineering Research Institute (CMERI), a public engineering research institution, has developed an innovative technology to aid hospital solid waste (HSW) disposal with low harmful emissions into the environment. With assistance from the Department of Science and Technology (DST), the facility utilizes plasma arc to process hospital solid waste completely, achieving a waste volume reduction efficiency of roughly 95%. This cutting-edge technology not only decreases the amount of garbage but also helps to keep the environment clean. It is being developed under the Waste Management Technologies (WMT) initiative, which encourages the creation of indigenous technologies to reduce environmental burden [32]. Additionally, the application of the new technologies Internet of Things (IoT) and Hospital based BMW management could provide location based information and waste management will create safe environment for health care workers and help in improving the ranking worldwide [33]. Recently, a new approach to managing BMW was developed with integration of communication and networking technologies that can improve monitoring and evaluation of BMW using Radio Frequency Identification (RFID) systems, a cost-effective and efficient method for tracking biomedical waste (BMW) in healthcare facilities [34]. Additionally, the surge of next COVID-19 waves and its impact on generation of BMW can be predicted with the previous data with the help of statistical forecasting methods. This would help in identification of hotspots and the policy-making will be made easy from safe handling and disposal of BMW.

## Conclusion

Sustainable management of BMW has been integrated with advanced online monitoring technologies and real-time tracking, providing critical assistance for safe handling and treatment and disposal planning. The accurate information derived from these technologies is essential for tracking BMW throughout its life cycle and for effective policy making. Detailed information on BMW generation and disposal at the ground level is particularly important for policy makers. Additionally, the mapping of waste treatment infrastructure can be used for future planning by utilizing available treatment locations. The use of the COVID-19BMW application to generate high-precision data has placed India at the forefront of real-time statistical monitoring of the generation and flow of medical waste. Proper policy maintenance and treatment methods can help prevent the spread of viruses, bacteria, and other diseases, thereby contributing significantly to environmental protection as well as the protection of healthcare facilities. The mapping technique facilitates the visualization of BMW generation at the district and state level. The use of Choropleth mapping, which utilizes a colour gradient to depict data, simplifies visualization and makes it easy to understand the nationwide generation of COVID-19 BMW. This technique also allows for the identification of changes between two temporal scales and is more attractive than traditional tabular forms of data representation.

## Data Availability

The numerical data related to health care waste and BMW is available in the public domain for access.

## Abbreviations

BMW: Bio-Medical Waste
COVID-19: Coronavirus disease
CMERI: Central Mechanical Engineering Research Institute
DST: Department of Science and Technology
GIS: Geographical Information System
HCW: Health Care Waste
HCF: Health Care Facilities
HSW: Hospital Solid Waste
IoT: Internet of Things
MoEFCC: Ministry of Environment Forest, and Climate Change
CBWTF: Common Biomedical Waste Treatment Facilities
INP: Information Not Provided
PPE: Personal Protective Equipment
RAT: Rapid Antigen Test
SDG: Sustainable Development Goals
TPD: Tonnes per Day
UT: Union Territory
WMT: Waste Management Technologies

## References

[1] Ramteke S, Sahu BL (2020) Novel coronavirus disease 2019 (COVID-19) pandemic: Considerations for the biomedical waste sector in India. Case Studies in Chemical and Environmental Engineering 2:100029. 10.1016/j.cscee.2020.100029 10.1016/j.cscee.2020.100029 PMC7395644 38620325

[2] Cheng YW, Sung FC, Yang Y, Lo YH, Chung YT, Li K-C (2009) Medical waste production at hospitals and associated factors. Waste Manage 29:440–444. 10.1016/j.wasman.2008.01.014 10.1016/j.wasman.2008.01.014 PMC7133359 18359619

[3] World Bank (2003a) Health Care Waste Management at a glance. https://web.worldbank.org/archive/website01213/WEB/0__CO-74.HTM.

[4] Elsheekh KM, Kamel RR, Elsherif DM, Shalaby AM (2021) Achieving sustainable development goals from the perspective of solid waste management plans. J Eng Appl Sci 68:9. 10.1186/s44147-021-00009-9

[5] Chand S, Shastry CS, Hiremath S, Joel JJ, Krishnabhat CH, Mateti UV (2021) Updates on biomedical waste management during COVID-19: The Indian scenario. Clinical Epidemiology and Global Health 11:100715. 10.1016/j.cegh.2021.100715 10.1016/j.cegh.2021.100715 PMC9393250 36032559

[6] Parivesh (2022) Bio Medical Waste (Management and Handling) Rules, 1998. https://parivesh.nic.in/writereaddata/ENV/HSM/note26.pdf.

[7] MPCB (2016) Bio Medical Waste Management Rules, 2016. https://www.mpcb.gov.in/waste-management/biomedical-waste. Accessed 16 Feb 2022.

[8] WHO (2018) Health-care waste. https://www.who.int/news-room/fact-sheets/detail/health-care-waste.

[9] MoHFW (2020) Environmental and Social Management Framework for India COVID-19 Emergency Response and Health Systems Preparedness Project. https://www.mohfw.gov.in/pdf/EnvironmentalandSocialManagementFrameworkforindiaCOVID19EmergencyResponseandHealthSystemsPreparednessProjectP173836.pdf.

[10] India.gov.in (2022) India national portal of India. https://www.india.gov.in/india-glance/profile. Accessed 10 Jan 2022.

[11] World Bank (2003b) Health Care Waste Management in India Lessons from Experience. http://web.worldbank.org/archive/website01291/WEB/0__CO-80.HTM.

[12] Economic survey of Maharashtra 2020–21. Directorate of Economics and Statistics, Planning Department, Govt. of Maharashtra. https://mahades.maharashtra.gov.in/files/publication/ESM_2020_21_Eng_Book.pdf.

[13] Datta P, Mohi G, Chander J (2018) Biomedical waste management in India: Critical appraisal. J Lab Physicians 10:006–014. 10.4103/JLP.JLP_89_17 10.4103/JLP.JLP_89_17 PMC5784295 29403196

[14] Wolf MJ, Emerson JW, Esty DC, de Sherbinin A, Wendling ZA, et al. (2022). Environmental Per-formance Index. New Haven, CT: Yale Center for Environmental Law & Policy. epi.yale.edu. https://epi.yale.edu/downloads/epi2022report06062022.pdf.

[15] Sarkodie SA, Owusu PA (2021) Impact of COVID-19 pandemic on waste management. Environ Dev Sustain 23:7951–7960. 10.1007/s10668-020-00956-y 32863738 10.1007/s10668-020-00956-y PMC7447614

[16] Ankit KA, Jain V, Deovanshi A, Lepcha A, Das C, Bauddh K, Srivastava S (2021) Environmental impact of COVID-19 pandemic: more negatives than positives. Environ Sustainability 4:447–454. 10.1007/s42398-021-00159-9 10.1007/s42398-021-00159-9 PMC7896832 38624614

[17] IFC (2021) Innovation in manufacturing personal protective equipment toward sustainability and circularity. https://www.ifc.org/wps/wcm/connect/industry_ext_content/ifc_external_corporate_site/manufacturing/resources/innovation+in+manufacturing+personal+protective+equipment.

[18] WHO (2022) Fortune favours the prepared: Fixing the COVID-19 waste problem to build back better and tackle climate change. https://www.who.int/news-room/feature-stories/detail/fortune-favours-the-prepared-fixing-the-covid-19-waste-problem-to-build-back-better-and-tackle-climate-change [Accessed on 26 Feb 2022]

[19] WHO (2022) Global analysis of healthcare waste in the context of COVID-19: status, impacts and recommendations. Geneva: World Health Organization; 2022. Licence: (CC BY-NC-SA 3.0 IGO). https://apps.who.int/iris/rest/bitstreams/1406822/retrieve.

[20] CPCBa (2020) User manual Android mobile application & web application: COVID19BWM COVID-19 Biomedical Waste Tracking App. Version 1.0 [Accessed on 26 Feb 2022]

[21] Bhar A, Biswas RK, Choudhury AK (2022) The influence of COVID-19 pandemic on biomedical waste management, the impact beyond infection. ProcIndian Natl Sci Acad. 10.1007/s43538-022-00070-9

[22] Das AK, Islam MdN, Billah MdM, Sarker A (2021) COVID-19 pandemic and healthcare solid waste management strategy – A mini-review. Science of The Total Environment 778:146220. 10.1016/j.scitotenv.2021.146220 10.1016/j.scitotenv.2021.146220 PMC7932852 33711590

[23] Rajabifard A, Paez, D, Foliente G (2021)The Role and Value of Geospatial Information and Technology in a Pandemic. In COVID-19 Pandemic, Geospatial Information, and Community Resilience: Global Applications and Lessons, 1st ed.; Boca Raton: CRC Press; 2021. pp. 3–10.

[24] CDC (2022) Types of thematic maps. https://www.cdc.gov/dhdsp/maps/gisx/resources/thematic-maps.html. Accessed 20 Feb 2022

[25] wikigis.com (2022) Quantile. https://wiki.gis.com/wiki/index.php/Quantile

[26] MPCB (2020) Bio-Medical Waste Annual Report 2020. https://mpcb.gov.in/sites/default/files/biomedical-waste/BMWAnnualReport2020MPCB03082021.pdf.

[27] MPCB (2021) Bio-Medical Waste Annual Report 2021. https://mpcb.gov.in/sites/default/files/biomedical-waste/reports/MPCB%20BMW%20Annual%20Report%202021-02082022.pdf.

[28] Nor Faiza M, Noor Artika H, Yusof M (2019) Health Care Waste Management and Sustainable Development Goals in Malaysia. J wast bio manag 18–20. 10.26480/jwbm.01.2019.18.20

[29] Singh N, Ogunseitan OA, Tang Y (2022) Medical waste: Current challenges and future opportunities for sustainable management. Crit Rev Environ Sci Technol 52:2000–2022. 10.1080/10643389.2021.1885325

[30] Ahuja A, Bhaskar S (2020) Coronavirus Pandemic Exposes Broken System Of Bio-medical Waste Management; Experts Discuss The Issue And Solutions. https://swachhindia.ndtv.com/coronaviruspandemic-exposes-broken-system-of-bio-medical-waste-management-experts-discuss-the-issue-and-solutions-49427/.

[31] Chisholm JM, Zamani R, Negm AM, Said N, Mahmoud M, Abdel daiem MD, Akrami M, (2021) Sustainable waste management of medical waste in African developing countries: A narrative review. Waste Manage Res 39(9):1149–1163. 10.1177/0734242X211029175 10.1177/0734242X211029175 PMC8488638 34218734

[32] dst.gov.in (2023) New technology can help disposal of hospital waste through electric arc-plasma. https://dst.gov.in/new-technology-can-help-disposal-hospital-waste-through-electric-arc-plasma

[33] Ramaa A, Nagendra Guptha C, Subramanya K, Patil A, Joshy N, Balakrishna P, Shettannavar S, Vithyathil A (2020) IoT Enabled Biomedical Waste Management System. RV Journal of Science Technology Engineering Arts and Management 1(1). http://rvjsteam.rvce.edu.in/pdf/RVJ06.pdf.

[34] Wawale SG, Shabaz M, Mehbodniya A, Soni M, Deb N, Elashiri MA, Dwivedi YD, Naved M (2022) Biomedical Waste Management Using IoT Tracked and Fuzzy Classified Integrated Technique. Human-centric Computing and Information Sciences 12:401–414. 10.22967/HCIS.2022.12.032

